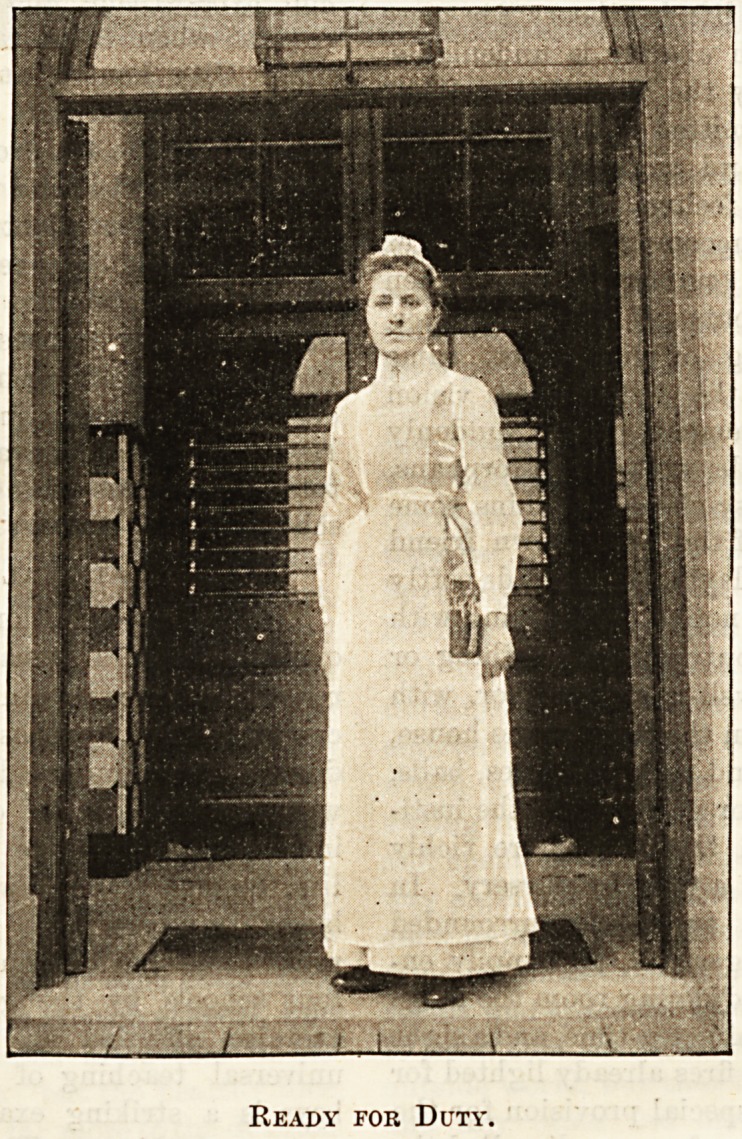# The Hospital.—Christmas Appeal Supplement

**Published:** 1896-12-19

**Authors:** 


HER MAJESTY THE QUEEN.

Victoria R I.

*By permission, from a photograph by W. & D. Downey, Ebury Street, London, S.W.*

### CHRISTMAS APPEAL SUPPLEMENT—ILLUSTRATED.

### Her Majesty the Queen.

We have much pleasure in to-day presenting our readers, who are drawn from every part of the British Empire, with a portrait of H.M. Queen Victoria, of Great Britain and Ireland, Canada, Australia, South, West, and East Africa, and Oceana, and Empress of India. We have it on the authority of an illustrious personage that the photograph from which the plate has been reproduced is quite the best which has ever been taken of the Queen. It is, therefore, an interesting and valuable souvenir, which we have no doubt will be prized as it deserves. It possesses an additional interest because the position is one chosen by the Queen, and on the table at which she is sitting is placed a portrait of the late Prince Consort, Albert the Good, whose memory is still honoured by the people of this country, who owe him so much in many ways.

It is only by reading history that it is possible to appreciate the changes that have occurred in the sixty years during which our gracious Queen has occupied the throne and has held the reins of government over this mighty empire. With all due loyalty we offer to Her Majesty our respectful congratulations on the eve of entering the year in which the sixtieth anniversary of her accession will take place. At the same time we must congratulate each other that we, our fathers and grandfathers, and, in many cases, our great grandfathers, have lived at such an era as to have partaken of the benefits which have fallen to the nation during a reign so long, so well ordered, and so beneficent. In saying this we do not think merely of the material prosperity of the nation, but look rather to those social changes which show how in every rank of life people have tended in their actions to reflect the virtues of their Queen upon the throne, and to emulate her good example.

The Royal example, always a power in the land, has been for the last sixty years a power for good. It may please the malcontents who see nothing good in the present day to look back to some mystic age when "the rich man helped the poor man, and the poor man loved the great." They do not read history, these worthy folks, or they would know that class hatreds were stronger long ago than now, that destitution was commoner and more keen, and acts of violence more frequent. They would know that never before this age was philanthropy so active or so ordered, that never before did the rich desire so earnestly to help the poor, and never before did the strong seek so systematically to help the weak. And they would understand that this great social change has been helped in no small degree by the influence and the example of the honoured lady who for sixty years has sat upon the British throne.

As mother, wife, and Queen, we have honoured her. As Queen, wife, and mother has she held out her hand in help and support to the numerous charities of the empire. Of Her Majesty's private charities we have no right here to speak. She has "done good by stealth" to those who would blush to receive help openly, and the charity which Her Majesty has chosen to do secretly it would not become us to drag to light. But of her public recognition of the philanthropic movements of the day it is both our pleasure and our duty to speak openly, and with loyal appreciation. It is not every applicant for Royal patronage to whom it can fitly be extended, even though it be asked for in the name of charity, and the way in which the Queen has bestowed not only her gifts, but that patronage which is so powerful in drawing gifts from others, should excite admiration both for its extent and its discrimination.

We have before us a list of sixty hospitals and dispensaries which are carried on under the direct patronage of Her Majesty. We have also a list of the ninety other charities of which the Queen is patron. And in these long lists we see not only the extent to which Her Majesty has interested herself in public charities, but we note how largely the hospitals and dispensaries figure among the institutions of which she is a patron.

In another column we show the progress which has been made by these charities, both in income and good works, during the past sixty years.

This is an advance in charity and good works which speaks well for the growth not only of the prosperity and giving power of the country, but also of the growth of that willingness to give which has been so characteristic of a reign in which Royal sanction and Royal patronage have always been extended to good works.

In this long list we find charities of the most varied description. Institutions connected with the Army, the Navy, and the Church; convalescent homes, orphanages, and benevolent funds; Masonic institutions, and such organisations as the Hospital Sunday Fund; and everywhere these institutions, which have received Her Majesty's gracious patronage, hold it not merely as an honour but as a responsibility, their managers desiring to make the charities under their control truly worthy of the patronage of a Sovereign at once sympathetic and just. To every corner of the realm, then, does the sceptre of mercy reach, to honour some institution or organisation of help and charity, to distinguish it by a noble title, and with the distinction to impress more deeply on those who guide it the motto of honour—*Noblesse oblige.*

Many of these Institutions have, no doubt, received the Royal patronage, because they are connected with the public services or with the National Church with which the Queen stands in special and official relations; others, perhaps, because of her infinite pity for even the weakest of her subjects, for while not only those professions which are most intimately associated with the national honour, and every occupation which contributes its share to national prosperity can boast that the Royal hand has pointed it out, by its patronage, as worthy of the support of the charitable, even those who are no honour to any nation, those sad outcasts of lust whose existence is one of the shadows of civilisation, may be helped by the thought that for more than a century pure women, queens whose ermine is unstained, have held out to them the hand of pity and help as soon as they have shown that earnest repentance which we associate with the name of "the woman who was a sinner."

But there are others in the long list of charities which boast of Her Majesty's patronage which we cannot help thinking were chosen for reasons which appealed to the woman as much as to the Queen. When in 1852 Queen Victoria, at that time surrounded by her own young family, became the Patroness of the Hospital for Children in Great Ormond Street, was it not because the mother's heart uttered the desire to help children who suffer, all the more that they come from homes where the parents cannot call in that experience and skill of doctors and nurses which are always at the command of the palace? In so far as the Queen can help, they are equally at the command of the poor. Just a century before, in 1752, Queen Charlotte had honoured with her name a hospital intended to receive poor women of honourable antecedents in their hour of trouble. Queen Victoria took up the charity her grandmother had founded, and became in turn its patron. In Scotland, also, there are hospitals which can say that to them the Queen has shown by her patronage her sympathy with the trials and anxieties of the wife and mother, a proof to all the world that beneath the imperial purple beats the heart of simple womanhood. To the afflicted ones whom the lack of one or other of the senses unfits to earn their living in ordinary ways, charity instinctively goes out, and therefore it is natural to find the Queen a patron of numerous institutions for the blind, not merely asylums and hospitals, but agencies for so educating the blind that they may compete on terms as equal as possible with their seeing brethren. The deaf and dumb also have their share of her consideration, and indeed it would be difficult to point to any conspicuous ill that flesh is heir to that is not represented in Her Majesty's list of charities.

Readers of THE HOSPITAL can never forget the interest shown by Her Majesty in the sick poor. The foundation of the noble association of the Queen's Jubilee Nurses proves how deeply Her Majesty appreciates trained care in sickness, and how earnestly she desires to extend that care to all her people. Indeed, there is no worthy form of charity to which in one way or another the Queen has not extended a helping hand. It was the glory of her ancestors to lead their people in war, but it will remain the special distinction of Queen Victoria that she has been "a leader and commander to her people" in the long warfare against disease, ignorance, and all forms of misery—a warfare whose end is not yet, but which is every year being carried on with more success, and is every year enlisting for its service a larger army of the earnest, the pure-souled, and the unselfish among her subjects.

### TO THE QUEEN.

REVERED, beloved, O you that hold
  A nobler office upon earth
  Than arms, or power of brain, or birth
Could give the warrior kings of old.

Take, Madam, this poor book of song;
  For tho' the faults were thick as dust
  In vacant chambers, I could trust
Your kindness. May you rule us long,

And leave us rulers of your blood
  As noble till the latest day!
  May children of our children say:
"She wrought her people lasting good;

"Her Court was pure; her life serene;
  God gave her peace; her land reposed;
  A thousand claims to reverence closed
In her as Mother, Wife, and Queen;

"And statesmen at her Council met
  Who knew the seasons when to take
  Occasion by the hand, and make
The bounds of freedom wider yet

"By shaping some august decree,
  Which kept her Throne unshaken still,
  Broad-based upon her people's will,
And compass'd by the inviolate sea!"

—*Tennyson.*

### Sixty Years of Voluntary Charity.

On page 23 will be found a complete list giving the years of foundation and the names of hospitals and other charities of which Her Majesty the Queen is the patron. The majority of these charities were founded previous to the Queen's accession to the throne in 1837.

### Hospitals and Dispensaries.

We have made an analysis of the accounts of the institutions patronised by the Queen, and of the reports of work done during the years 1837 and 1895 respectively. So far as the hospitals and dispensaries are concerned, these figures will no doubt afford food for much thought. Thus, the number of beds in the hospitals in 1837 amounted roughly to 2,300, and in 1895 they had increased to 3,331. These beds were utilised by about 28,000 in-patients in 1837, and by only 36,514 in 1895. These last figures are very surprising, especially when we compare them with the growth of the out-patient returns. Thus only 72,674 out-patients were admitted to relief by the institutions in question during the year 1837, but in 1895 the enormous number of 344,091 out-patients were treated at the hospitals apparently without fee or payment of any kind. Whatever else these figures may indicate, they certainly reveal an immense amount of benefit, which must have been pretty largely distributed amongst a large number of people who have it in their power to support as well as to bleed the hospitals. Dealing first with the ordinary income, it appears that the revenue from this source, exclusive of legacies, in 1837 was £58,258; whereas, in 1895, this revenue had increased more than threefold, or to £183,589. Having regard to the fact that, although the available hospitals' beds have greatly increased, the number of in-patients has not increased by more than 30 per cent. during the period dealt with, we are justified in concluding that the multiplication of out-patients has entailed an enormous growth in the cost of maintaining medical institutions, a fact which those who have abused the hospitals most by accepting relief at their hands when they could well afford to pay a doctor should take to heart, and make a point of recognising in cash during the year 1897. The ordinary expenditure was £61,704 in 1837, and it increased to £219,961 in 1895; there was therefore a deficiency between ordinary income and ordinary expenditure in 1895 of £36,000. Fortunately the extraordinary income was large enough to wipe out the deficiency thus created, and to provide a sum so large as to leave a balance in favour of the institutions. It is very remarkable that as the out-patients have grown out of all proportion to the needs and necessities of the people, so the extraordinary expenditure has grown (from £5,942 in 1837 to £26,800 in 1895, the largest increase in proportion of any of the items we have dealt with). Extraordinary income is chiefly derived from legacies, which amounted in 1837 to less than £10,000, but had increased in 1895 to £79,142, showing that the dead hand has not lost its power nor the tenacity with which it underpins and sustains the great voluntary charities of the British Empire.

### General Charities.

This list includes orphanages, army and navy benevolent funds, the Royal Literary Fund, blind, indigent, destitute, lifeboat, provident, and other institutions. It will not be necessary to do more in this section than to indicate that the growth in income is keeping pace with the expenditure, and that both have increased enormously during the last sixty years. The ordinary income in this group, exclusive of legacies, amounted to £101,051 in 1837, but had grown to £311,962 in 1895. On the other hand, the ordinary expenditure, which was £97,186 in 1837 now (1895) amounts to the enormous sum of £330,196 roughly; a deficiency is thus shown for 1895 of something like £19,000 per annum. This has been provided out of the extraordinary revenue derived from legacies which has risen from £18,018 in 1837 to £74,536 in 1895. We are not able to account precisely for the interesting fact that the extraordinary expenditure of this class of charities was £2,400 less in 1895 than in 1837.

There can be no doubt from the figures we have given that the voluntary charities have greatly prospered, and have become enormously popular under Queen Victoria. As the population has increased and the demands upon the charities have grown, so the contributions of the benevolent have grown with the needs of the charities. This is as it should be, no doubt; but it is disheartening to find that a careful examination of the facts proves to the hilt that, as in 1837 so in 1895, the proportion of the population who contribute anything regularly to the hospitals or to any charity of any kind is so small as to be relatively insignificant when compared with the whole population of these islands.

### Some Novel Facts.

The statement we have ventured to make in the preceding paragraph has been so often laughed at and declared to be unsustainable by evidence that we think it well to take the opportunity of utilising some most interesting facts and figures compiled by Mr. Alexander M. Chance from the seventeen reports of the General, Jaffray, and Queen's Hospitals and fourteen amalgamated charities in Birmingham for the year 1894. Exclusive of donations and of contributions from the Hospital Saturday and Sunday Funds, the number of personal subscribers to the charities of Birmingham in 1894 was 4,343, out of a total population of 478,000 people. Assuming that on an average the number of each family amounts to six persons, it will be seen that the personal subscribers to the Birmingham charities only represent something like one in twenty of the population. Of these personal subscribers, 62¼ per cent. gave to one hospital only, 17·7 per cent. to two institutions, 7¾ per cent. to three institutions, less than 4 per cent. to four per institutions, rather more than 2¾ per cent. to five institutions, 1½ per cent. to six institutions, about 1¼ per cent. to seven institutions, and so on until we come to one person, the sole representative apparently in Birmingham of systematic giving, who is an annual subscriber to every one of the seventeen charities. It would appear, further, from the Birmingham figures that the older the charity the larger the number of subscribers and the greater its popularity. Next in order of popularity comes the Children's Hospital, and we imagine that within certain well-defined limits this represents the prevailing feeling to be met with everywhere. The percentage of personal subscriptions was as follows: 51

per cent. contribute one guinea per annum; 21 per cent. between one and two guineas; nearly 18 per cent. from two to five guineas; 6·64 per cent. from five to ten guineas; 1¾ per cent. from ten to fifteen guineas, up to nearly ¼ per cent. who give from £30 to £50 per annum, and another ¼ per cent who give over £50 per annum each.

We take it that Mr. Chance's figures are eloquent of much. They prove that, unless or until a person has learned to think more of others than of himself, he ignores charity, and refrains from giving anything to alleviate the sufferings or necessities of his less fortunate townsmen or countrymen. The condition of affairs at Birmingham we have reason to believe is not worse but rather better than that of many other great towns. Compared with London, although the regular supporters of the charities at Birmingham are, comparatively speaking, so limited as to strike wonder to the heart of the inquiring foreigner, who is immensely impressed by the circumstance that some of the largest and most attractive buildings occupying the best sites in many English towns bear upon their face the words, "supported by voluntary contributions," the first impression as to Birmingham, small in proportion though the giving population undoubtedly is, is that the percentage which it bears to the total population is infinitely larger, comparatively speaking, than is the case in the metropolis of the empire. No doubt the voluntary charities have greatly prospered and increased in influence, efficiency, and usefulness during the sixty years of Her Majesty's reign. In our judgment, however, the proportion of the non-givers to givers is so out of proportion to what it ought to be, as to stir the hearts of all thinking men and women who desire to see England as a nation vibrating with love, joy, hope, and charity, through the awakened benevolence of all classes, including the greatest as well as the humblest members of the community.

### WITHIN THE HOSPITAL: FRIENDS FROM OUTSIDE.

We are going to visit the chief general hospital of a large city, and our way lies through narrow, sunless streets. It is well, for the sake of comparison, that we should learn something of how the poor live and die in their own homes. Let us, therefore, like old-time writers, take you by the hand. We will follow that district nurse. The coster who bids her a cheery good day is, like ourselves, on his way to the hospital. The nurse is entering a mean-looking house across the street, and we will go with her. We grope our way up a ricketty wooden staircase and along a noisome passage. It is difficult to find words to describe the squalid sick-room. It is the old story of sudden ruin caused by illness among those who find it difficult enough to make ends meet at the best of times. The room has been stripped of everything save a huge four-poster—which may well be large, as it "sleeps" a family of six at night. Even the odd pieces of delf — most cherished chattels of the poorest poor!—are gone from the dresser. The fire smouldering in the grate is small, yet the air of the room is suffocating. A glance at the window shows that the chinks in the sash have been pasted over "to keep out the draught." On the bed lies a young woman from whose eyes the light of life is fast fading. Her mother sits by her on an upturned packing-case. She seems dazed by grief and want of sleep, and takes but little notice of the nurse until the latter tries to open the window. Then she is up in arms to prevent the admission of fresh air.

Presents for the Patients.

But it is time we continued our journey. Here we are on the main thoroughfare, with the hospital on our right. It is visiting day, and the friends of patients are gathering at the gate. The barrow of our old friend the coster is drawn up by the roadside, and many stop to buy some little present. The proprietor is genial and communicative. "Oh, bless yer, yes," he explains, in answer to a question, "I makes a speciality of it; but it's only a part o' my trade. This ain't strict business, you see. My little girl was in there for nigh three months, an' w'en they're buyin' things for the kids I'm not partickler to a ha'porth." On joining the waiting crowd we find much discussion going on. An old lady is detailing a dream she had during the previous night, in proof of the fact that her "man" is going to regain his sight. The talk, indeed, is mostly about the "cases" of their friends within. One cadaverous youth, however, waxes autobiographic, and gives a verbose clinical lecture on his "bronchial toobs." He relates how he went to a chemist and got a mixture that had "no more taste than water"; but, later on, having fallen on evil times, applied at the outdoor department of the hospital, and was supplied with a mixture that acted "like a miracle." "It *was* a good bottle," he adds, reflectively; "the first dose nearly took my breath away." A diversion is caused by a poor woman fainting. For want of a few pence she has walked a long distance to see her child. Forthwith a man rushes into the street and returns in an astonishingly short time with some brandy. The woman, who is reviving, smiles feebly, and pretends to sip the stimulant. It is now nearly three, and expectant glances are directed towards the closed door. It opens as the hour strikes, and the crowd surges in. Eager, however, as everyone is to reach the wards, the woman who

The Rush to Enter.

fainted is accorded the first entry. Coming in from the noisy street, the peace that reigns throughout the great building is striking. The scattering visitors feel the influence, and lower their voices. Let us follow them at our leisure.

We stand at the door of a female ward. The big chamber looks bright and cheery. The snow-white linen on the beds stands out against the darker walls and floor. Here and there palms and other plants are tastefully grouped. The blazing fires, reflected in the polished floor, add a last touch of comfort to the scene. The patients are mostly sitting up in bed to welcome their friends. The colour of excitement is in their cheeks, and many of them look ridiculously well. Already tongues are going apace, for there is much to tell, and the women have their own ways of telling it. Let us look around with the eye of understanding. The buxom girl in the nearest bed is graciously accepting a bunch of flowers from a shy young man, while her mother looks on with grim approval. On the other side is a fair-haired woman who has been face to face with death for many a day, but is now slowly recovering. She is silent, holding her mother's hand. She is strangely like the patient we saw dying in that dreadful den—but how different her surroundings! On the other side of the ward is a garrulous old lady, who is criticising the treatment of the eminent physician, whose "case" she is. Nevertheless she is improving wonderfully, and her two dutiful sons exchange furtive winks when she sighs for a decoction of some herb which used to grow near her Scotch home. That refined-looking patient at the far end has the saddest of histories. Her white face grows whiter when her son tells her that his father has not come because he is "ill" again. Still she smiles, and bids him be a good lad, and keep his father from "company," till she is strong enough to return home and "get things to rights." And so each bed has its tragedy or comedy; but time flies, and we must be moving.

While still in the corridor we can hear treble voices broken by rippling laughter—and here we are in the children's ward. Can these little people be of the same stock as those who sit by their cots? Sister will tell you that ward life produces a marked moral improvement in the case of children. No doubt the impression is transient, but it is pleasant to think that a child's stay in hospital may be a revelation of a higher life bearing fruit long after. Here is a little boy strapped to a splint, so that he has to spend much of his time lying almost on his face. At home his life would have been one of continuous wretchedness and pain. Now he is fairly happy, and hugs an ugly Chinese doll with a wan smile. His face flushes with pleasure when Sister tells his mother how good and patient he is. Further down the ward are two children who have no visitors. One of them appears to feel her isolation keenly. It is her birthday, and ever since early morning she has been waiting with feverish excitement for her father and mother to arrive with the gigantic doll which has been the talk of the ward for a week. Sister tells us that when they did not appear with the other visitors she seemed too hurt to cry; but the tears came when another patient's mother spoke to her kindly. When Sister sought to comfort her with toys even the well-beloved slate had lost its attraction. She has now ceased crying, but, although all hope is gone, her eyes still involuntarily seek the door of the ward. The other unvisited child—a flaxen-haired little girl—is philosophic. She sits bolt upright in bed, and examines the other patients' friends with critical blue eyes. "Mother has not come to-day," a nurse remarks as she is pass-

My Birthday! Father and Mother have not come.

ing. "I 'spect she thinks I's dead" is the quaint reply; but even as she speaks her face breaks into dimples—her mother is at the door.

We have still to visit a male ward. It is quieter than the others. The men, are the breadwinners of the family, and many of them are anxiously consulting with their wives as to the best way of keeping the wolf from the door until they are about again. That is a striking family group by the window. The man's resolute, careworn face tells something of his life. After receiving a good education, he was apprenticed to a highly-specialised trade which is now almost extinct. Being an expert, he held his own for a long time, but at last even his work ceased to find a market. For two years he lived in dread of the workhouse. Then a relative unexpectedly left him a few hundred pounds. This money he invested in some small works on the understanding that he should keep the books, and act as under-manager. At the outset the business was in a tottering condition, but in a few years his energy once more placed it on a sound basis. Then his sight began to fail, partly owing to eye-strain while following his original trade. He kept this secret, and often worked at his books far into the night. One day the works—uninsured—were burned to the ground: and Fate had a still greater blow for the half blind and now penniless man, for, while trying to save some papers in the office, the metal fittings of a falling beam tore his arm and severed one of the main nerves. His left arm was consequently paralysed. Of his subsequent life we know little, until he turned up at the outdoor department of the hospital. He was admitted, operated upon, and is now rapidly recovering. His sight, too, has wonderfully improved under proper treatment. So, as he says, the hospital has made a new man of him.

Other histories as interesting we could relate, but the bell rings, and it is time to go. On the landing we meet an aged gentleman whom we noted reading a newspaper to a crony in one of the wards. As we linger to see him safely down the stairs he informs us, by way of introduction, that he has known better days, and was once a schoolmaster. "Hospitals are grand institutions," he goes on to remark. "Look what this one has done for my friend—pulled him through a serious illness, and ensured his pension to his children until they are old enough to hold their own. It is possible to do a man harm in aiding him—but hospitals take him when he is helpless and hopeless, and put him on his feet again. Long may they flourish!"

A Typical Group within the Wards.

To which—and having seen and heard for yourself, we hope you will agree with us—we say, Amen.

J. B. and M. D.

### Choosing a Charity.

Our Christmas supplement is probably unique in this, that the advertisements are, without exception, pertinent to the matters in which readers of The Hospital are interested. It contains the appeals of the various hospitals, stating, in the words of their own officials, their actual wants at the present time. Those who at this season are in doubt where to bestow that charity, which is to an Englishman so essential and necessary a feature of an English Christmas, cannot do better than cast their eyes over the appeals which appear in this week's supplement, and, having done so, to fill in the accompanying *Bankers' Form* in favour of such objects as may most appeal to their sympathy.

### Another Field of Charity.

### THE MAKING OF CITIZENS.

The crowded streets of the City at mid-day have justly been considered one of the most notable sights in London. Many foreigners visiting England for the first time have been struck not only with the vast and ceaseless streams of traffic, but with the earnest purpose visible in each face; and some have insisted, wondering at this feature in our national characteristics, on the note of painful care evident in almost every case as underlying the immediate purpose of the hour. It is the greed for money is the common accusation. Rather, we believe and know, that in the vast majority of cases the anxiety is that of the man whose income depends on his own exertions, and who is conscious that when health or life fail those most dear to him will stand face to face with want. The extent to which this gnawing unrest about the future of wife and children tortures the worthiest members of the middle class is little understood. Men are reticent concerning such cases. With every effort and thrift the unnumbered crowd of minor professional men, clerks, subordinate officials, and those enaged in small commercial and agricultural enterprises can but insure their lives for a sum offering the barest subsistence for the widow, and quite incapable of meeting the prolonged strain of educating children. Nothing more natural than that at the slightest hint of breaking health the problem, "What will become of them after my death?" becomes a serious aggravation of the malady, and that it should not unseldom be known to drive the sufferer to the last extremity of despair. But it is essentially an English trait (may we not be proud to recognise this in the festival of Him who taught us to value the children!) that men who have felt this gnawing care themselves, who have seen it justified by the death of comrades and the desolation of homes, should have borne it in their minds, not for themselves, but for others, as they pressed onwards and upwards in the ranks of the prosperous, and that such men should in the end have found the worthiest destination for their accumulated wealth, and their highest joy in making provision for the destitute orphans of their fellow-workers. It is for the workers themselves that the impulse has come for the doing of this noble work, and glad furtherance has been found for it among the ranks of those who can afford their aid. We propose to give some account of four institutions, representing an initial outlay of some quarter of a million sterling, embracing the care of eighteen hundred children, and supported at an annual cost of over £50,000.

That a hard-working, thrifty man should be consoled in his dying moments by the knowledge that his helpless sons and daughters will be safely cared for and honourably educated, is in itself no slight thing for which to give money. But the responsibility is great and the reward very high when all the consequences of this act are followed to the end in view. What awaits these children unaided? For some a precarious education may be provided by the generosity of friends. We say precarious, because how often it happens that after a year or two of such support the benefactor dies, or is himself impoverished, and the promised aid is perforce withdrawn. For the majority nothing else can be expected but a violent lapse in the social scale, involving a retrogression as unjust to the honest life-work of the parents as it is cruelly bitter to the child and dangerous to the community. Untrained, uncared for, unfitted for any but the overcrowded ranks of the most unskilled labour, the future of these orphaned children, accustomed every one, be it remembered, to the comforts of a gentle home, would indeed be dark if in the hour of their loss the hand of Charity, wise and strong, were not stretched out to help.

The four schools to which we allude are: The British Orphan Asylum, the London Orphan Asylum, the Infant Orphan Asylum, and the Orphan Working School. The end in view at each of these institutions is the making of good citizens, and how effectually it is attained may be judged by the fact that at each and all the applications from well-known firms for the lads at the conclusion of their term of training are more than can be met. Boys, as a rule, are considered fit for work at the age of fifteen, and they pass then to the offices of lawyers, merchants, and bankers, and to wholesale or retail commercial houses, for which the well-ordered routine of their school life has served in a special manner to adapt them. After this start in life their future is in their own hands. The vast majority secure a comfortable and independent livelihood; many rise to important positions of responsibility, and some, like Bishop Hill of the Orphan Working School, attain to unusual distinction. All over the world men are scattered at this moment helping to maintain the honour of the Empire, who were once in danger of falling far behind as stragglers in the race of life for want of a father's care.

The future of girls trained in schools such as these is necessarily less assured than that of the boys, excepting always those where the aim is to prepare for domestic service, as in this case the girls are always in request among the members of the committee and friends of the institution. In the higher grade schools girls are not as a rule retained after the age of fifteen unless in the position of pupil teacher, and while recognising fully that the institution life beyond that age may be actually harmful, it cannot be denied that they are seldom fitted to earn their own livelihood so early, and that a critical time remains to be tided over before the launch in life can be made. There are, however, nearly always friends who can help for a year or two, and the institutions themselves make grants to the most promising pupils to assist them in completing their course of training. The routine, as in the case of the boys is found eminently to fit them for clerkships, and many pass after a year or two into the post office, the telegraph service, and other offices where lady clerks are employed. Many more are engaged in teaching, and in one way or another nearly all succeed in maintaining themselves. We could wish, however, that all who exert themselves to get a child into an orphanage would remember that their interest in it should not end there. An orphan child should engage their continued countenance and friendly offices during the whole period of residence in the orphanage, and, indeed, until it is fairly started in the world and able to shift for itself. Otherwise vicarious charity may do harm rather than good to those who shirk the responsibilities which they should lovingly take upon themselves.

### Four Great Orphan Schools.

The Infant Orphan Asylum.

### The British Orphan Asylum, Slough.

This institution was founded in 1827 for the benefit of "the children of those once in prosperity." The number at present in the school are—Boys, 100; girls, 86; junior boys in a separate house, 20. The school-house, in which both boys and girls are accommodated in separate wings, has been adapted from an hotel of some importance in the old posting days, and the wide staircases and lofty rooms overlooking the Thames Valley and Windsor Castle preclude the possibility of any lack of air and light. Beautiful grounds surround the house, and fine playgrounds for both boys and girls, cricketing field, fives-court, asphalted tennis-court, and swimming-bath adjoin. The pupils remain until the age of fifteen, and are expected to pass the Cambridge preliminary or junior examinations before leaving. The observer cannot fail to be struck by the absence in both the boys and girls' school of the usually inevitable institution element. The impression is of a well-ordered private school, where the children are individuals as well as parts of an harmonious system. This may be partly due to the facts that comparatively small numbers are received, that the total annual cost of each child (£38) is a good deal above the average at such schools, and that longer holidays—three months altogether in the year—are allowed to the children; but the healthy tone of the school must be attributed far more to the devoted labours of those responsible for its welfare during many years past. The British Orphan Asylum has been more than commonly fortunate in enlisting the personal services and sympathies of men eminent in education and philanthropy. There is an admirably constructed junior school for twenty little boys, aged from seven to ten, under the motherly care of the lady superintendent, Miss Caithness. The advantage to these little lads of a separate and homelike household immediately on their separation from home does not need to be pointed out. The infirmary accommodation in two separate cottages for ordinary and infectious cases is admirable.

### The London Orphan School, Watford.

Fifty acres of ground are occupied by this school, which offers unique advantages in promoting the physical welfare of the 500 children (300 boys and 200 girls) sheltered under its roof. The building is an imposing one, and the magnificent dining-room and spacious chapel, with its memorial windows, are well calculated to impress all visitors with the dignity of the institution and the munificence of its friends. The object is the maintenance, clothing, and education of fatherless children who are respectably descended. Both boys and girls prepare for the preliminary and junior Cambridge examinations, and the successes attained at the latest examinations attest to the efficiency of the teaching. The buildings are divided into separate blocks, each connected, however, with a central hall, where all dine together. The girls occupy a large wing, but the boys are accommodated in separate houses, each holding no more than fifty, and under the control of a lady matron. Each house contains two dormitories for twenty-five lads, separated by the matron's room, which contains two curtained alcoves, which overlook them. There is in addition a large sitting-room, where the boys can prepare their lessons and spend their evenings. The arrangement is in every way excellent, and cannot fail to have a refining influence impossible of attainment where vast dormitories must accommodate sixty or seventy children, and playrooms find space for hundreds together. We were glad to learn that the lads were not entirely cut off from the outside world, but that four monitors from each house, and any other lad against whose name stands the password of "excellent," were allowed the privilege of taking walks together on half-holidays. The only matters for regret are—first, that the girls, for whom the association of large numbers is even far less salutary than for boys, were not separately housed also in the original design; and, secondly, that two of the houses built for the boys should be forced to remain permanently empty for want of funds to maintain a hundred extra children. And here it may be mentioned, for the benefit of those who are willing and able to advance this good work, that the yearly cost of each child averages little over £30, so that a matter of £3,000 a year would fill both empty houses with youthful life and energy and set a hundred new little citizens on the road to industry and prosperity. All the food supply, sanitary playgrounds, infirmary, and other arrangements are excellent.

### The Infant Orphan Asylum, Wanstead.

This institution, as its names implies, has deviated from the usual rule of receiving orphan children only after the age of seven, and offers a shelter to the fatherless from their earliest infancy. It is intended for the benefit of those whose fathers have filled "a respectable position in society," and offers a sound middle-class education to, in all, 600 childen until they reach the age of fifteen. These 600 are accommodated in the beautiful grey stone building in the following separate departments: Senior boys, 230; junior boys, 86; senior girls, 170; junior girls, 86; nursery children, 28. The situation of the school in the open ground adjoining Epping Forest, with ample space for outdoor games, is entirely in its favour. No part of the institution is likely to be more keenly scrutinised than that designed for the reception of infant children. Much as one must grieve at the thought of institution life in theory for mere babies, the need for some such charity is undeniable, and is pitifully exemplified by the fact that at Wanstead "infants have been admitted at the tender age of six weeks, both fatherless and motherless." Let it be frankly admitted that a feeling of strong prejudice against the system accompanied the writer to the very door of the nursery. But when that was opened, and the shouts of merry laughter, with the unsubdued trampling of little feet, came echoing out into the hall, the half-formed vision of heavy faces and listless movements melted suddenly away. Here were no poor little well-drilled orphans, but a boisterous company of rosy-cheeked urchins, some clutching boldly at the legs of the well-known friend who was acting escort, others laying their heads softly on his coat to secure a kindly notice, all beaming with pleasure, and ready to laugh outright at anything or nothing. Up and down the well-worn corridor, with cheerful windows looking out on the front of the house, they were playing with carts and rocking-horses, balls, and playthings in abundance, for no friend of the institution forgets the babies, and they are more richly endowed with toys than many a wealthy nursery. In the pleasant playroom beyond nurse was surrounded by a little group of those younger still, all happily engrossed in their toys. In an adjoining room the mugs were being laid for the approaching tea-time, and a sight of the airy sleeping-room, with fires already lighted for the night, and cosy cots, with special provision for the very youngest ones on each side of nurse, dispelled the last lingering fear of any unnatural element in the life of these little ones. But how do they fare when they leave this happy nursery life? After four they pass on to the junior boys and girls' schools respectively, where they begin lessons, and where in their own classrooms and playrooms they have a domain still quite apart from the main body of scholars. Here they get accustomed to discipline and routine, and are prepared for the final step, which at the age of eight launches them into the big school world. The total annual cost of each child is £29.

### The Orphan Working School, Haverstock Hill.

Here accommodation is found for 246 boys and 96 girls. The building is admirably adapted to the purpose for which it is intended. The class-rooms are lofty and well planned and have been, with one small exception, approved as regards light, air, and space by the Government expert since the school has recently passed under direct Government inspection. The dormitories are all thoroughly airy and good, and are fitted throughout with hot-water pipes. There is ample playground space both for boys and girls, and an excellent swimming bath. The pupils are for the most part children of clerks, retail traders, skilled mechanics, and gentlemen's servants. The education afforded till the age of fourteen, when the boys go out to situations and the girls are taken into training for domestic service, is now identical in most respects with that given at the Board Schools. To pass the seventh standard, for which the senior lads are now preparing, is test of a sound and thorough grounding in all subjects likely to be required in commercial situations; to these subjects the boys voluntarily add type-writing and shorthand. They are in great request when they leave school in large retail and other City houses, and maintain the reputation of the school in all parts of the world. They are a well-grown, athletic set, proud of their cricketing skill and football victories, and they have earned by their manly and upright conduct an unusual degree of trust, fully justified by results from those in charge. The girls, receiving an education similar to that of the boys, are passed into the house after thirteen for practical training in house and laundry work, and cooking. There is a junior school, situated in Hornsey Rise, and a small convalescent home at Margate. The total annual cost of each child in the orphan working schools is £26.

### Material Considerations.

The healthy condition of the 1,800 children in these orphanages is a remarkable testimony not only to recent advances in sanitary science, but to thoughtful care exercised by those in charge over the inmates down to the minutest details of their lives. Everyone who has had charge of children is aware that little indiscretions or wants, which pass unnoticed in family life, become fraught with the gravest dangers where large numbers are gathered together under one roof. We have been specially struck in all these four schools by the generous provision for outdoor exercise afforded to girls as well as boys. The universal teaching of swimming to girls as well as boys is a striking example of this new and better system of things. The kindly tone of modern education, which inspires interest in the work undertaken and affection towards the teacher, has abolished the old listlessness which used to be so marked a feature of institution life. Preparation for examinations or for the visit of the inspector gives a point to the daily routine and acts as a helpful stimulus to both teachers and pupils. Again, outside school there is now the emulation of matches undertaken against other schools, or of new occupations or hobbies, encouraged by the teachers, who are themselves not too far removed in sympathy from their pupils to be drawn into the current of the prevailing interest. In this way individuality finds a vent, general ability or special talent makes its mark, and the love of employment is cultivated as a habit likely to endure.

Each and all of these institutions may be safely commended to the generosity of the public.

### The Hospital Nurse.

At Christmas time, when kind thoughts should go out to all our fellow-creatures, one class is especially worthy of our sympathy and consideration, viz., the nurses; for they are doing all the year the very thing which is so appropriate at Christmas time that to many it would almost seem peculiar to that season—helping the sick, comforting the suffering, and spending their time, their energies, and too often their health, in doing good to others. To the hospital nurse Christmas is not the time of pleasure and festivity which it is to people in other walks of life, unless, indeed, as is so often the case, she takes an actual pleasure in hard work, for Christmas in hospitals is a time of work in a far greater degree than many people think. The decoration of the wards, the arrangements for the visitors, the carol singing, the Christmas trees, and all the little entertainments which are got up to break the monotony of hospital life to those who lie in bed, and to make them feel that although sick and unfortunate they are not forgotten; all these things add greatly to the work of the nurses, partly by throwing upon them unaccustomed labour, partly by rendering the ordinary daily routine of the wards more difficult.

And yet how cheerfully and with what alacrity they join in all the plans for the patients' good! How they go about with hammers and tin-tacks! How they weave garlands and make festoons! How brightly and laughingly they climb ladders and put up wreaths and crosses and pretty texts, when they ought to be lying down and resting their weary backs! And then how cheerfully they devote themselves on Christmas Eve, and on the actual Christmas Day, to making their patients happy, giving up for their sake the family gathering, the jolly meeting which only can take place once a year, when brothers come from far parts of the country and sisters bring up baby nephews and nieces to be kissed, and there is feasting and happy talk, and cares are laid aside, and home is home. All this, the very essence of the social and family side of Christmas, is given up without a murmur by the hospital nurse in order that she may continue in her chosen work and pass the festive season in nursing the sick, in cheering the despondent, and perchance in soothing the last moments of the dying. So when the cup goes round and crackers fly, when jokes resound and sweet nonsense is being whispered in the ears of blushing maidens, let us not forget the absent sister, the gentle nurse, who is keeping her quiet vigil in the darkened ward. The hospital nurse may be sure that there is one place where she is remembered kindly, and where her memory is kept warm. Patients may feel bu a doubting, scanty, and impersonal sort of gratitude to the hospital that has taken them in; the surgeon, by whose skill a man's life has been saved, may remain to him but a hazy memory, as an occasional visitor among a crowd of students, perhaps even as a man whose visits were rather dreaded; but to his nurse his gratitude is personal and real, and many a time has it happened that the image of the nurse who has looked after him in an illness in his youth has remained to a man through a rough, hard life of struggle for subsistence, almost the sole memory of kindness, simplicity, and disinterestedness.

Ready for Duty.

In many a home in London where Christmas means but a poor and meagre feast, and where little ones with thin faces gather round a scantily-provided board, kind thoughts of thankfulness go out towards the nurse, who they know is caring for the absent one, the brother or the sister, the mother, may be, or the father and the breadwinner, who is laid up in the great hospital, and whose fate, to their minds, at any rate, appears to lie in the hands of the gentle nurse who soothed them and was kind when they left their dear patient in her charge. Such people know, as we know, but as we fear too many people fail to recognise, the enormous benefit which a patient suffering from serious illness receives by being removed from the ordinary poor man's home to a well-appointed hospital. The dwellers in tenements know better than anyone else can know the impossibility of obtaining proper treatment for the sick in places where there is not even proper accommodation for the healthy. It is not idleness or indifference that makes the poor glad to send their sick to hospital, but a complete recognition of the fact that it is in hospital alone that they have any chance of proper treatment, and in hospital alone that they have a reasonable likelihood of restoration to health. Yet the going is a wrench. The one figure that gives comfort to the mother, may be, for having left her child within the house of suffering, is that of the gentle nurse. She does not ask questions, sh( does not give minute directions, nor offer papers to be signed; all she does is to talk kindly, and to promise that she will look after the little fellow. But kindness and personal sympathy go far, and the mother is content; and many a night when she dreams about her boy, a vision of the gentle nurse intrudes itself as of a guardian angel.

### Under the Patronage of the Queen.

The following is a list of the British and Irish hospitals, dispensaries, lunatic asylums, nursing institutions, convalescent homes, orphanages, institutions for the blind and deaf, and numerous other charities of which the Queen is patron, together with the date when they were founded. This list is likely to be useful and a matter of interest to many charitable people who would no doubt like to have an opportunity of making a special gift to some or all of them during the sixtieth year of Her Majesty's reign. We have taken great pains to make this list as complete as possible, but we should be very glad to hear from the authorities of any institution which has been inadvertently overlooked and is so omitted, if such there be.

### ADMINISTRATIVE AND COLLECTIVE SOCIETIES.

1873 Metropolitan Hospital Saturday Fund.

### LONDON—HOSPITALS.

1820 Charing Cross Hospital.
1848 City of London Hospital for Diseases of the Chest.
1868 East London Hospital for Children.
1850 Establishment for Gentlewomen.
1867 French Hospital.
1765 General Lying-in Hospital.
1841 Hospital for Consumption, Brompton.
1852 Hospital for Children, Great Ormond Street.
1839 King's College Hospital.
1745 Middlesex Hospital.
Disp. 1783, H. 1883. Miller Hospital and Royal Kent Dispensary.
1859 National Hospital for Paralysed and Epileptic.
1833 North London, or University College Hospital.
1752 Queen Charlotte's Lying-in Hospital.
1816 Royal Ear Hospital.
1828 Royal Free Hospital.
1814 Royal Hospital for Diseases of the Chest.
1816 Royal Hospital for Women and Children.
1804 Royal London Ophthalmic Hospital.
1838 Royal Orthopædic Hospital.
1816 Royal Westminster Ophthalmic Hospital.
1733 St. George's Hospital.
1845 St. Mary's Hospital.
1821 Seamen's Hospital Society.
1719 Westminster Hospital.

### LONDON—DISPENSARIES.

1757 Royal Maternity Charity.
Royal South London Dispensary.
1774 Westminster General Dispensary.
1770 Royal General Dispensary.

### PROVINCIAL—HOSPITALS.

1840 Birmingham, The Queen's Hospital.
1855 Bournemouth National Sanatorium for Consumption.
1719 Cambridge, Addenbrooke's Hospital.
1866 Guildford, Royal Surrey County Hospital.
1752 Manchester, Royal Infirmary.
1791 Margate, Royal Sea Bathing Infirmary.
1846 Portsmouth, Royal Portsmouth, Portsea, and Gosport Hospital.
1869 Preston and County of Lancaster Royal Infirmary.
1839 Reading, Royal Berkshire Hospital.
1868 Richmond, The Royal Hospital.
1827 Salford, Royal Hospital.
1812 Scarborough, Royal Northern Sea Bathing Infirmary.
1868 Ventnor, Royal National Hospital for Consumption.
1836 Weymouth and Dorset County Royal Eye Infirmary.
1816 Weymouth Royal Hospital.
Disp. 1818, Inf. 1857 Windsor and Eton Royal Dispensary and Infirmary.
1736 Winchester, Royal Hants County Hospital.

### PROVINCIAL—DISPENSARIES.

1809 Brighton, Hove, and Preston Dispensary.

### SCOTLAND—HOSPITALS.

1739 Aberdeen, Royal Infirmary.
1858 Aberdeen, Hospital for Incurables.
1859 Edinburgh, Royal Hospital for Sick Children.
1793 Edinburgh, Royal Maternity and Simpson Memorial Hospital.
1883 Glasgow, Royal Hospital for Sick Children.
1791 Glasgow, Royal Infirmary.
1782 Montrose Royal Infirmary and Dispensary.
1838 Perth, County and City of Perth Royal Infirmary.

### IRELAND—HOSPITALS.

1791 Belfast, Royal Hospital.
1832 Dublin, City of Dublin Hospital.
1753 Dublin, Meath Hospital and County of Dublin Infirmary.

### LUNATIC ASYLUMS.

1546 London, Bethlehem Royal Hospital.
1847 Earlswood, Asylum for Idiots.
1849 Manchester, Royal Lunatic Hospital.

### NURSING INSTITUTIONS.

1835 Royal Derby and Derbyshire Nursing and Sanitary Association.

### RELIGIOUS SOCIETIES.

1823 Colonial and Continental Church Society.
1837 Society for Promoting the Employment of Additional Curates.
1881 Mission to Deep Sea Fishermen.

### CONVALESCENT HOMES.

1840 Metropolitan Convalescent Institution.
1881 Morley House Convalescent Home.
1825 Stoke Newington Invalid Asylum.

### ORPHANAGES, HOMES, AND CHARITIES.

1855 Army and Navy Pensioners' Employment Society.
1837 Booksellers' Provident Institution.
1827 British Orphan Asylum.
1811 Cheltenham Orphan Asylum.
1851 Choir Benevolent Fund.
1881 Church of England Incorporated Society for Providing Homes for Waifs and Strays.
1862 Church of Eng. Temperance Society.
1818 City of London General Pension Soc
1885 Civil Service Benevolent Fund.
1749 Clergy Orphan Corporation.
1846 Domestic Servants' Benevolent Institution.
1758 Female Orphan Asylum.
1802 Friendly Female Society.
1838 Gardeners' Royal Benevolent Institution.
1875 Girls' Friendly Society.
1885 Gordon Boys' Home National Memorial.
1843 Governesses' Benevolent Institution.
1827 Infant Orphan Asylum.
1818 Incorporated Church Building Society.
1887 Irish Distressed Ladies' Fund.
1702 Ladies' Charity School.
1794 Licensed Victuallers' Permanent Relief Fund.
1869 Liverpool Seamen's Orphan Institution.
1807 London Female Guardian Society.
1813 London Orphan Asylum.
1758 Magdalen Hospital.
1870 Metropolitan and City Police Orphanage.
1829 Metropolitan Benefit Societies' Asylums.
1812 National Benevolent Institution.
1884 National Society for the Prevention of Cruelty to Children.
1816 National Society for Promoting the Education of the Poor, &c.
1758 Orphan Working School.
1788 Philanthropic Society for the Reformation of Criminal Boys.
1827 Printers' Pension, Almshouses, and Orphan Asylum Corporation.
1840 Provident Clerks' Benevolent Fund.
1844 Ragged School Union and Shaftesbury Society.
1858 Railway Benevolent Institution.
1844 Reedham Orphanage.
1805 Refuge for the Destitute.
1882 Rowland Hill Benevolent Fund.
1860 Royal Agricultural Benevol. Institn.
1864 Royal Albert Orphan Asylum.
1702 Royal Asylum of St. Anne's Society.
1839 Royal British Orpham Society, Devonport.
1815 Royal Caledonian Asylum.
1851 Royal Cambridge Asylum for Soldiers' Widows.
1822 Royal Female Philanthropic Society.
1774 Royal Humane Society.
1790 Royal Literary Fund.
1798 Royal Masonic Institution for Boys.
1788 Royal Masonic Institution for Girls.
1851 Royal Medical Benevolent College.
1824 Royal National Lifeboat Institution.
1739 Royal Naval Benevolent Society.
1884 Royal Provident Fund for Sea Fishermen.
1864 Royal School for Daughters of Officers of the Army.
1857 Royal Society for the Assistance of Discharged Prisoners.
1824 Royal Society for the Prevention of Cruelty to Animals.
1836 Royal Society for Protection of Life from Fire.
1829 Sailors' Orphan Girls' School & Home.
1836 St. Marylebone Almshouses Institution.
1839 Shipwrecked Fishermen and Mariners' Royal Benevolent Society.
1844 Society for Improving the Condition of the Labouring Classes.
1698 Soc. for Prmtng. Chrstn. Knowledge.
1860 Society for the Relief of Distress.
1823 Society for the Relief of Distressed Widows.
1818 Society for the Suppression of Mendicity.
1855 Soldiers' Daughters' Home.
1885 Travellers' Aid Society.
1885 Soldiers and Sailors Families' Association.
1860 Temporary Home for Lost and Starving Dogs.
1863 United Kingdom Beneficent Society.
1850 Wolverhampton Orphan Asylum.

### Institutions for the Blind and Deaf.

1868 British & Foreign Blind Association.
1834 Indigent Blind Visiting Society.
1838 London Society for Teaching the Blind to Read, &c.
1843 National Blind Relief Society.
1863 Royal Blind Pension Society.
1872 Royal Normal College for the Blind.
School for the Indigent Blind.
1791 Liverpool School for the Indigent Blind.
1793 Edinburgh Royal Blind Asylum and School.
1840 Royal Association in Aid of the Deaf and Dumb.
1810 Institution for the Education of the Deaf and Dumb, Edinburgh.

### SWEET CHARITY'S GUIDE TO CHRISTMAS GIVERS.

### GENERAL HOSPITALS.

**Charing Cross Hospital**, Agar Street, West Strand, W.C.—This institution is situated in the midst of some of the most crowded thoroughfares in the metropolis, and has, therefore, to provide for a greater number of accidents than probably any other hospital of its size. All such cases are immediately admitted without delay or difficulty. 25,000 patients were relieved during the past year, more than two-thirds of which were cases of accident or emergency. £2,000 is required to enable the hospital to close the year without incurring any fresh liability. A special appeal is also being made under the auspices of the Duke of Saxe-Coburg and Gotha for £100,000 to place the hospital on a sound financial basis, and to provide accommodation for the largely-increased work thrown upon the hospital nowadays. Secretary, Mr. A. E. Reade.

**German Hospital**, Dalston, N.E.—This hospital contains 125 beds, and admits all who are conversant with the German language, without distinction of nationality or creed, as well as accident and emergency cases. Superintendent and Secretary, Mr. H. Gülich.

**Great Northern Central Hospital**, Holloway Road, N.—This hospital is entirely free to the sick poor, no letters of recommendation being required. The new hospital, completed in 1894, is one of the most beautiful and perfect in London, and every detail has been carried out in accordance with modern scientific knowledge. Over 1,400 in-patients and 2,400 out-patients are treated annually. The hospital is unendowed, and the reliable income is quite inadequate to meet the necessary expenditure—over £8,000 being required annually from voluntary sources. There is also a debt of £3,000 on the building fund. Annual subscriptions and donations may be sent to the Secretary, Mr. Lewis H. Glenton-Kerr.

**Guy's Hospital**, London Bridge, S.E.—Guy's is too old a friend of the public to need much to be said in its favour; but so long as it has closed wards and is crippled by poverty it is a reproach to the metropolis. The net income of the hospital derived from its landed estates remains at £20,000 per annum less than it was before the agricultural depression, with no present prospect of improvement. There is left, therefore, only an assured income of £20,000 to maintain the 500 beds now open, which cannot be kept up at a less cost than £43,000 per annum. In response to the appeal of H.R.H. the Prince of Wales, £170,000 has been received during the present year for investment, but the greater part of the deficiency in income can only be met in the future by liberal contributions from the public. Funds are also required to reopen 100 beds still remaining vacant, and for which there is a pressing demand. Treasurer, Mr. H. Cosmo Bonsor. Superintendent, Dr. Perry.

**Hampstead Hospital**, Parliament Hill Road, N.W.—This hospital was established in 1882 as a general hospital for paying patients. In 1894 the constitution of the hospital was altered, and the general wards were made free, an out-patient department being also opened. This change now supplies a want long felt of a local general hospital. The number of in-patients in 1895 was 270, besides minor accidents and casualties. Of these 168 were free cases. There were also nearly 1,000 attendances in the out-patient department. These numbers have very considerably increased during the present year. The expenditure far exceeds the income, the deficiency last year being £800. There is also a heavy mortgage on the building which the council are most anxious to reduce. Donations and new annual subscriptions are urgently needed to carry on the work. Secretary, Mr. R. A. Owthwaite.

**Italian Hospital**, Queen Square, Bloomsbury, W.C.—Though primarily established for the treatment of Italians, this hospital admits persons of all nationalities, over 10 per cent. of the in-patients and 5 per cent. of the out-patients treated last year having been English. The present building is quite inadequate, and most unsuitable for the work which the hospital now does, and the committee are therefore appealing for funds to rebuild. A gentleman, by whose benevolence the work of the hospital has mainly been carried on since its establishment, has given the freehold of an adjoining house as a site for additions, and it is hoped that funds will be forthcoming to enable the rebuilding of the hospital in the near future. Secretary, G. Ferrari.

**King's College Hospital**, Portugal Street, Lincoln's Inn, W.C.—2,717 in and 26,175 out-patients were treated in 1895, in addition to 700 poor married women attended during confinement in their own homes. Warden, Rev. N. Bromley.

**London Homœopathic Hospital**, Great Ormond Street, W.C.—This hospital has a well-earned reputation for efficiency and careful management. The new building, opened in 1895, has been in full working order during the past year. The number of in-patients will exceed 1,000, and the number of out-patients 14,000. The committee are making an urgent appeal for the remaining £5,000 due to the New Building Fund, in order that the hospital may start the coming year entirely free from debt. To maintain the present hospital means an additional annual expenditure of £2,000 a year, and to relieve the committee of all anxiety as to being able to keep open the beds at their disposal, it is imperative that new annual subscriptions for this amount should be forthcoming. Treasurer, Viscount Emlyn, 7, Princes Gardens, S.W. Secretary-Superintendent, Mr. G. A. Cross.

**London Hospital**, Whitechapel Road, E.—This is the largest voluntary hospital in this country. From this fact, and from the zealous manner in which the work is carried on, we make no doubt that the public will give largely to its funds, in testimony of their appreciation of the enormous value of what is being done there for the poor of East London. It includes special departments under eminent medical men for the treatment of all classes of disease, the number of beds devoted to children being greater than those to be met with in most children's hospitals. It is in serious want of funds, as the committee depend on voluntary contributions for £40,000 a year to enable them to maintain the 650 beds which are daily occupied by urgent cases. House Governor and Secretary, Mr. G. Q. Roberts, M.A.

**Metropolitan Hospital**, Kingsland Road, N.E.—The need for this hospital in the poor and densely populated districts in the midst of which it is situated, is shown by the fact that both the in and out patients have been steadily on the increase for several years past, the numbers treated last year being, in-patients, 971; out-patients' attendances, 90,776. In spite of this good work the expenditure last year exceeded the income by £1,504, leaving a debt of over £6,000 incurred during the past three years. The charity is much crippled by lack of funds, and several beds have had to be closed, leaving only 66 now available for in-patients although there is accommodation for 160. Urgent cases have therefore constantly to be refused admission, and unless money is forthcoming at once it will be necessary to still further reduce the number of beds. Secretary, Mr. C. H. Byers.

**Middlesex Hospital**, Mortimer Street, W.—This hospital contains 321 beds; the in-patients for last year numbered 3,404, and the out-patients 42,922 (total, 46,326); the income from all sources, including legacies and an amount of £866 specially contributed towards the cost of building a new cancer wing, was £36,856, and the expenditure (including the investment of the £866), £38,929; deficiency, £2,073. The cancer wards are a distinguishing feature of the hospital, and their extra nursing, costly treatment, and unlimited dietary add largely to the expenses of the hospital. A scheme, which has received the approbation of H.R.H. the Prince of Wales, is on foot to extend this department by removing it from the main body of the hospital and building a new wing, at a cost of £12,000, of which £4,000 is still needed. There is a convalescent home containing 45 beds and isolation wards at Clacton-on-Sea. Secretary Superintendent, Mr. F. Clare Melhado.

**North-West London Hospital**, Kentish Town Road, N.W., was founded in 1878, and is the only institution of the kind in the north-west district. It has 53 beds, and last year there were 683 in-patients and 21,227 out-patients. Secretary, Mr. Alfred Craske.

**Poplar Hospital for Accidents**, Blackwall, E.—The recent extensions at this hospital have greatly increased the work, consequently further subscriptions and donations are very necessary. The statement made in a recent report that during the whole year 1895 accidents were treated in the hospital at the rate of four for every hour of every day, will bring home to everyone the great benefit the institution is to the district. This fact, coupled with its energetic management, should command hearty support. Chairman, the Hon. Sydney Holland. Secretary, Lieut.-Colonel Feneran.

**Royal Free Hospital**, Gray's Inn Road, W.C.—Having no endowment, this hospital is entirely dependent for support on the subscriptions of its governors and the voluntary donations and bequests of its friends. It admits into its wards about 2,000 poor sick persons annually, besides administering advice and medicine to more than 33,000 out-patients who resort to it, not only from the crowded courts and alleys in its immediate neighbourhood, but from all parts of London and the suburban districts. The relief thus afforded is effected at a cost of about £10,000 per annum, while the reliable income of the charity from annual subscriptions and other sources does not exceed £3,000, so that the large balance of £7,000 has to be raised by means of constant appeals to the public benevolence. Secretary, Mr. Conrad W. Thies.

**St. George's Hospital**, Hyde Park Corner, S.W.—This institution contains 351 beds, and during 1895 treated 4,191 in-patients, 15,450 out-patients, 12,824 casualties, and 427 maternity cases were treated. Secretary and Superintendent, Mr. C. L. Todd.

**St. Mary's Hospital**, Paddington, W.—Very heavy work is thrown upon this institution owing to the large area of scattered poor which it has to assist. It is not so well supported as it should be, in spite of or perhaps because of its many wealthy neighbours. The board of management, therefore, urgently appeal for further support. The special wants of the hospital are: (1) £5,000 to pay current quarter's bills, bankers; (2) donations for the endowment of beds and cots; and (3) £65 in new annual subscriptions to the Maternity Fund to provide a nurse for the maternity. The hospital is free, and no urgent case is refused admission. Secretary, Mr. Thomas Ryan.

**St. Thomas's Hospital**, Westminster Bridge Road, S.E.—Early last year a special appeal was issued with the object of raising a sum of £100,000 to enable the wards closed for lack of funds to be opened. Funds sufficient to open two of these wards have been received, and the wards have since February, 1896, been used for patients, but about £70,000 is still needed to make up this Sustentation Fund. Subscriptions to Mr. Wainwright, the Treasurer, at the hospital.

**Seamen's Hospital Society, "Dreadnought,"** Greenwich, S.E.—This hospital was established in the early part of the century and has now relieved over 373,000 sick, injured, and shipwrecked sailors. Secretary, Mr. P. Michelli.

**University College Hospital**, Gower Street, W.—The North London or University College Hospital is mainly dependent upon voluntary contributions, the income on which it can rely being only £7,000, whilst the necessary annual expenditure is very nearly £19,000, and the present debt exceeds £15,000. Owing to this the committee have reluctantly decided to close 50 of the 210 beds on March 1st next, unless by that time sufficient means are found to enable them to carry on the present work of the hospital without increasing the debt. Secretary, Mr. Newton H. Nixon.

**West London Hospital**, Hammersmith Road, W.—Last year (1895) the beds were occupied by 1,730 patients, and the out-patient department was attended by 32,410 others. Secretary, Mr. R. J. Gilbert.

**Westminster Hospital**, Broad Sanctuary, S.W.—Out of an expenditure of £14,000, less than £3,000 is assured to this charity, so that about £11,000 has to be made up each year in subscriptions. The work of the hospital in 1895 included the treatment of 2,142 in-patients, 18,138 out-patients and casualties, and 299 lying-in women. Altogether nearly one and a half million patients have been cared for by this hospital since it was established in 1719. The hospital does an immense service to the country by training a large number of excellent nurses. Secretary, Mr. Sidney M. Quennell.

### PROVINCIAL HOSPITALS.

**The Birmingham General Hospital.**—This useful institution is continuing its work under difficulties until the completion of the fine building which is making rapid progress. Funds are most earnestly appealed for towards the building fund and general maintenance of the hospital. The expenditure far exceeds the assured income, and annual subscriptions are especially needed, seeing that they have decreased from £5,204 in 1893 to £4,966 in 1895. House Governor, Mr. Howard J. Collins.

**Birmingham and Midland Counties Sanatorium, Blackwell and Sutton Coldfield.**—This institution has recently been given a new home at Sutton Coldfield, the keeping up of which will add materially to their annual expenditure. Additional help is therefore needed. Secretary, Mr. E. J. Bigwood, 17 and 19, Colmore Row, Birmingham.

### SPECIAL HOSPITALS.

### CONSUMPTION.

**Brompton Hospital for Consumption and Diseases of the Chest**, S.W.—This hospital is well known as one where patients are well cared for, and every effort is made to brighten their clouded lives. At this time of the year the Christmas tree, from which each patient is supplied with a suitable present, is a source of great interest, and contributions towards this object are most acceptable. Of late years many subscriptions to the hospital have been withdrawn through failure of income and other causes; the committee are most anxious that the places of those who formerly subscribed should be filled up by other kind friends. Secretary, Mr. W. H. Theobald.

**City of London Hospital for Diseases of the Chest**, Victoria Park, E.—This hospital is situated in the East of London, where the diseases it treats are so common. About 1,000 in-patients and 15,000 out-patients are treated yearly. The average annual expenditure is more than £11,000, towards which only £370 of the income is assured. Assistance is much required to repay loan from bankers to meet current expenditure. Donations or annual subscriptions will be thankfully received by the Secretary, Mr. T. Storrar-Smith; or by the bankers, Messrs. Barclay and C

**North London Hospital for Consumption,** Mount Vernon, Hampstead, N.W., and Fitzroy Square, W.—This institution was thoroughly reorganised and remodelled some years ago, and has now become one of the most useful of its class; 400 in and 3,000 out patients are admitted each year. There is room in the hospital for 80 beds, but the present income does not admit of more than 60 being opened. Secretary, Mr. W. G. Farrance Bosworth.

**Royal Hospital for Diseases of the Chest,** City Road, E.C.—This is the oldest consumption hospital in Europe, having been founded by Her Majesty s father, the late Duke of Kent, in 1814. There are 80 beds, and last year 755 in-patients and 9,491 out-patients were treated. The expenditure exceeds £8,000, towards which there is an annual subscription list of £2,000, and dividends amounting to about £100. Two additional wards have recently been opened, and funds are urgently needed. Donations will be gratefully acknowledged by the Secretary, Mr. John Harrold, or may be sent direct to the Chairman, Mr. T. Andros de la Rue.

**Royal National Hospital for Consumption and Diseases of the Chest at Ventnor,** Isle of Wight, provides accommodation (including separate bedrooms) for 134 patients (83 men and 51 women). As the expenses exceed the assured income by £4,000, the committee appeal for additional annual subscriptions and donations. Secretary, Mr. Ernest Morgan, London office, 34, Craven Street, Charing Cross, S.W.

### LYING-IN.

**City of London Lying-in Hospital,** City Road, E.C.—Over 2,000 poor women were safely delivered either in the hospital or at their own homes during 1895. Only one woman died in the hospital during the year, a fact which is a sufficient proof that the antiseptic treatment is fully carried out. Funds are needed to carry on the general work of the hospital. Secretary, Mr. R. A. Owthwaite.

**Queen Charlotte's Lying-in Hospital,** Marylebone Road, N.W.—To carry on the work of the charity efficiently £4,700 is needed annually, towards which the only assured income amounts to £1,800. To provide for the continuous growth of the work of the charity an extension of the hospital has been resolved upon, for which, with many necessary improvements, and for building a new nurses' home, upwards of £12,000 is very urgently needed. Her Majesty the Queen has contributed £50 towards the Extension Fund. Donations to this special fund, as well as for general maintenance, are earnestly solicited and will be thankfully received by the hospital bankers, Messrs. Cocks, Biddulph, and Co., 43, Charing Cross, W., or by the Secretary (Mr. Arthur Watts), at the hospital.

### EPILEPSY AND PARALYSIS.

**Hospital for Epilepsy and Paralysis, and other Diseases of the Nervous System,** Regent's Park, N.W.—The last annual report recorded a diminished income from all voluntary sources except legacies. Secretary, Mr. H. Howgrave Graham.

**National Hospital for the Paralysed and Epileptic** (Albany Memorial), Queen Square, W.C.—The total number of beds provided at this hospital and its Finchley branch is 200, but the accommodation is still painfully insufficient to meet the requirements, as in a large majority of cases the patients are unsuited to general hospitals. The attendances of out-patients are upwards of 33,000 yearly. More than 1,500 different cities, towns, and villages have sent in patients. Besides the hospital for treatment, there is a pension fund for the incurables. The annual expenditure is nearly £15,000, of which more than £8,000 must be raised in benefactions. Director, Mr. B. Burford Rawlings.

**West-end Hospital for Paralysis, Epilepsy, &c.,** 73, Welbeck Street, W.—A special appeal is now being made to enable the committee of this institution to pay off a debt of £6,000 incurred in rebuilding. The hospital contains fifty beds. The total number of in-patients treated last year was 217, of whom 76 came from the provinces, and only 46 from the West-end of London. Treasurer, Mr. H. Alexander Dowell.

### CHILDREN.

**Alexandra Hospital for Children with Hip Disease,** Queen Square, Bloomsbury, W.C.—Hip disease is attended with great suffering, and without proper treatment it ends in the patient either dying from exhaustion or being crippled for life. The necessity for the hospital may best be shown by the fact that nearly three-fourths of its patients come from other London hospitals, the reason being that no general hospital can retain a patient for two or three years as is not infrequently done in this hospital. The hospital is absolutely unendowed, and depends solely upon voluntary contributions. Additional help, which is urgently needed to prevent the work being curtailed, will be gratefully received by the secretary, Mr. Stanley Smith.

**East London Hospital for Children and Dispensary for Women,** Shadwell, E.—If institutions such as St. George's and St. Mary's Hospitals suffer from absence of adequate support, it will readily be understood that the struggle for existence is much greater in such a neighbourhood as Shadwell, and it is for this reason that the committee of the East London Hospital for Children appeal to the more wealthy classes for funds to enable them to help those who are unable to help themselves. Secretary, Mr. Thomas Hayes.

**North-Eastern Hospital for Children,** Hackney Road, Shoreditch.—This hospital, placed in the midst of one of the most crowded districts of East London, does an excellent work amongst the children of the poor. Extraordinary efforts have been made in order to pay off its indebtedness (which had accumulated until it reached £4,500), and the institution is now free from debt. It must, however, soon again become involved unless increased support can be obtained. Secretary, Mr. T. Glenton-Kerr. Matron, Miss E. W. Curno. City office, 27, Clement's Lane, E.C.

**The Hospital for Sick Children,** Great Ormond Street, W.C.—Founded in 1852, with twenty beds, this was the first hospital solely devoted to the sick children of the poor. There are now nearly 200 beds at Great Ormond Street, besides 52 beds at the Convalescent Branch at Highgate. About 1,700 in-patients, besides about 22,000 new out-patients, have been treated in 1895. Annual subscriptions and donations have during the past couple of years shown a serious falling off, and £1,000 in new annual subscriptions and £500 in donations are asked for to meet the cost of maintenance. Secretary, Mr. Adrian Hope.

**Victoria Hospital for Children,** Queen's Road, Chelsea, S.W. (and Victoria Convalescent Home, Broadstairs).—Established at Chelsea in 1886 for the relief of the sick and suffering children of the poor, this unendowed hospital seeks further subscriptions to help it to carry on its work. The expenditure reaches £7,000 to £8,000 a year, and this has to be raised entirely by voluntary subscriptions. Secretary, Commander Blount, R.N.

### WOMEN.

**Chelsea Hospital for Women,** Fulham Road, S.W.—This hospital has 52 beds, and treats respectable poor women and ladies in reduced circumstances. Contributing in-patients pay 10s. 6d. to 42s. a week, according to their means. The hospital is entirely without endowment or reserve funds, and greatly needs legacies and new annual subscriptions. In connection with the hospital is a Convalescent Home at St. Leonards-on-Sea, which has 22 beds. Secretary, Mr. Herbert H. Jennings.

**Grosvenor Hospital for Women and Children,** Vincent Square, Westminster, S.W.—While the bulk of the out-patients is drawn from the poorer classes living in the neighbourhood, the in-patients are received from all parts of the country, and both residents and non-residents of London may well give the institution a generous support. The hospital, hitherto inadequate and ill-contrived, is now being reconstructed with modern improvements. The new out-patients' department is already open. Increased support is urgently needed. Secretary, Mr. W. E. Hartopp.

**New Hospital for Women,** 144, Euston Road, N.W.—Only a want of funds prevents the committee from providing the beds necessary to treat the numerous cases waiting for admission to this hospital. Each bed costs about £75 a year, and an appeal is made to generous friends to come to the aid of the committee by endowing a bed with an amount that would produce this annual sum. Secretary, Miss M. M. Bagster.

**Royal Hospital for Children and Women,** Waterloo Bridge Road, S.E.—486 in-patients and 8,033 out-patients were treated during 1895. The income amounted to about £4,326; the ordinary expenditure to £4,278; and the extraordinary expenditure to £67. Secretary, Mr. G. H. Southern.

**Samaritan Free Hospital for Women and Children,** Marylebone Road, N.W.—Some £7,000 per annum is required to maintain this hospital, of which only £1,750 can be relied upon in annual subscriptions. The committee would therefore like to see a great addition to the list of annual and life subscriptions, and earnestly appeal for Christmas and New Year's gifts. Bankers, Scott and Co., 1, Cavendish Square, W. Secretary, Mr. G. Scudamore.

**The Hospital for Women,** Soho Square, W.—This institution (founded in the year 1842) claims the distinction of being the first established in this or any other country, exclusively for the treatment of women's diseases. There are 60 beds in constant use, and, as the hospital possesses no endowment, funds for their maintenance are much needed. The committee very earnestly appeal for additional annual subscriptions not only to Londoners, but to the whole of the United Kingdom, from all parts of which patients are received. Secretary, Mr. David Cannon.

### MISCELLANEOUS—SPECIAL.

**Cancer Hospital,** Fulham Road, Brompton, S.W.—Since its establishment in 1851, about 43,000 persons suffering under the terrible scourge of humanity which it was designed to relieve have been treated at this hospital. Secretary, Mr. W. H. Hughes.

**Central London Ophthalmic Hospital,** Gray's Inn Road, W.C.—This hospital, which has treated over 277,000 patients during the fifty-two years of its existence, is in much need of funds. Secretary, Mr. J. G. Bryant.

**Dental Hospital of London,** Leicester Square, W.C.—This charity is in urgent need of help. The committee bought some houses for their new site, relying upon the rents the houses produced for a few years to accumulate money for building. Recently the houses have been condemned, and are being demolished; consequently, the authorities are compelled to begin building the new hospital. Such a proceeding must necessarily involve the charity in considerable indebtedness, towards defraying which help is most seriously asked. Secretary, Mr. J. Francis Pink.

**Lock Hospital and Home,** Harrow Road, W.—Rescue work, as well as the cure of the patients who present themselves for admission, is the aim of this institution, and the good work has only to be known to meet with the support it deserves. A considerable number of those that pass through the hospital are reclaimed from their evil lives, which is a great result. A debt of under £500 has to be cleared off, and £1,000 in fresh annual subscriptions are required to meet the regular needs of the institution, and prevent the recurrence of debt. Secretary, Mr. A. W. Cruikshank.

**London Fever Hospital,** Liverpool Road, Islington, N.—The resources of this hospital have been much taxed during the last three years owing to the prevalence of scarlet fever and diphtheria. The institution is dependent upon voluntary support, and donations and subscriptions will be gratefully received, especially as the alterations and additions to the hospital, now being carried out, and the building of a convalescent home, will tax to the utmost the resources of the institution. Secretary, Major W. Christie.

**National Orthopædic Hospital,** 324, Great Portland Street, W.—The hospital's pressing needs are first to see the end of a debt of £1,650 on the building, and to increase its list of annual subscribers to three times its present length. Secretary, Mr. H. J. Tresidder.

**Royal Eye Hospital,** St. George's Circus, Southwark, S.E.—In proof of the necessity for this hospital, and for funds to extend its usefulness, the committee quote the following figures, which show the new cases treated during the following years: 1860, 1,690; 1870, 3,486; 1880, 5,040; 1890, 6,076; 1895, 14,453. Secretary, Mrs. J. E. Cope.

**Royal London Ophthalmic Hospital,** Moorfields, E.C.—An urgent appeal is made by the board of management for funds in support of this institution. The expenditure amounts annually to over £7,000, whereas the certain income is about £2,000. The patients numbered in 1895 in-patients, 2,184; out-patients, 26,290. All patients are admitted free, and without letters of recommendation. Secretary, Mr. Robert J. Newstead.

**Royal Orthopædic Hospital,** 297, Oxford Street, W.—The majority of out-patients at this hospital come, as might be expected, from the Metropolitan districts, but in-patients are drawn from all parts of England, more especially from the Midland and Southern Counties. The hospital is, therefore, more than a mere local charity, and it relieves maladies with which the sufferer is born, and which, if not remedied, may condemn him to lifelong misery and make him a burden to his friends already in poor circumstances. Secretary, Mr. Tate S. Mansford.

**St. Luke's Hospital,** London, E.C.—The demands on this hospital and its Convalescent Branch at Ramsgate, during the year have been exceptionally heavy, thereby drawing very largely on its funds. The Committee are compelled to carry out extensive repairs to the building owing to its age, and they confidently appeal for help to this charity, which discharges a special work in treating acute mental diseases of the middle classes, for whom the State has made no adequate provision. Treasurer, Mr. Edward W. Nix. Secretary, Mr. W. H. Baird.

**Western Ophthalmic Hospital,** 155, Marylebone Road, W.—At present existing in a private house, quite unfitted for hospital purposes, and growing needs, the necessity for erecting new buildings has become urgent, and Sir Reginald Hanson, treasurer to the hospital, has issued an appeal for funds towards this desirable end. Help is earnestly asked for. Secretary, Captain Hastings Neale.

### A FEW OTHER CHARITIES.

**Association for the Oral Instruction of the Deaf and Dumb.**—This association was the first to publicly introduce into the United Kingdom the German or pure oral system for teaching deaf and so-called dumb children to speak *viva voce*, and to understand the spoken words of others by lip-reading. The expenses, which are very heavy, are met by voluntary contributions and fees, which fall short of what is needed by about £700 per annum, and at the present moment the association is in debt to its bankers to the amount of £3,500. Financial aid is most earnestly solicited, and cheques may be directed to the Secretary at the offices, 11, Fitzroy Square, W.

**Bethnal Green Free Library.**—This pioneer institution, which is freely open to all comers, has now been in existence for twenty years, and has just commenced its winter's work, viz., evening classes for technical instruction to the youth of both sexes at reduced fees. The chief feature of the establishment is, of course, the library, which is the third largest in London. The poverty in the surrounding neighbourhood compels the committee to make an urgent appeal for funds to meet outstanding liabilities. Contributions will be thankfully received by the Librarian, at the Bethnal Green Free Library, E., or by the Treasurer, Mr. F. A. Bevan, 54, Lombard Street, E.C.

**City of London Truss Society,** 35, Finsbury Square, E.C.—At the present time about 10,000 of both sexes and all ages are treated annually. The increase in the number of female patients since separate entrances for male and female patients have been provided has been considerable, and should lead to an increased number of lady subscribers. The steady growth of the society in recent years is proved by the fact that more patients have been treated during the last twenty-nine than in the fifty preceding years. Secretary, Mr. John Whittington.

**Homes of Hope,** 4, 5, and 6, Regent's Square, Gray's Inn Road, W.C.—These homes have now for thirty-five years been steadily working for the restoration of fallen and the protection of friendless young women. During that period 3,562 young women and girls have been admitted. Lack of funds prevents the work being more extended, and the committee appeal most earnestly to the affluent and wealthy for the wherewithal to continue their useful work. Secretary, Mr. W. Hornibrook.

**Hospitals for Women in India.**—The Zenana Bible and Medical Mission has now three hospitals under qualified lady doctors doing most useful work amongst the women of India, at Lucknow, Benares, and Patna, the attendances at which last year amounted to 66,600. They carry on an extensive work, besides educational, medical, and religious, amongst the female population of India, and are in need of constant help to continue and extend their labour. General Secretary, the Rev. A. Cavelier; Hon. Finance Secretary, Mr. W. T. Paton. Offices, 2, Adelphi Terrace, W.C.

**London Orphan Asylum, Watford.**—Five hundred children are now profiting by the advantages given through this old-established charity, no less than one hundred and eleven having been admitted during the past year. Almost every locality in the United Kingdom has been represented in the list of those who have been cared for at this institution. Donations towards the necessary income, almost entirely derived from voluntary sources, will be thankfully received by the Secretary, Mr. H. C. Armiger, 21, Great St. Helen's, E.C.

**London Schools Dinner Association**—Established in 1889, to provide cheap or free meals for necessitous children attending the public elementary schools of London, this association is doing an excellent work. The annual income now averages about £1,500, but it is reckoned that double that sum is necessary to meet the needs of the children. Secretary, J. A. Spalding, 37, Norfolk Street, Strand, W.C.

**Mary Wardell Convalescent Home for Scarlet Fever,** Stanmore, Middlesex.—The only home for convalescents from scarlet fever. The demand on its forty beds is great, and expenses are heavy. Funds are greatly needed to meet the yearly expenses, and also to pay off a debt unavoidably incurred in renovating the home, after more than nine years' work. Subscriptions to Miss Mary Wardell.

**Metropolitan Drinking Fountain and Cattle Trough Association.**—This is the only society which provides free supplies of water for the many thousands of men, women, and children, besides horses, cattle, sheep, and dogs that are daily toiling through our streets. It depends entirely upon voluntary contributions, which are urgently needed, as the year ended March 31st, 1896, closed with liabilities amounting to about £4,600. Secretary, Mr. M. W. Milton, 70, Victoria Street, Westminster, S.W.

**National Food Supply Association,** Memorial Hall, Farringdon Street, E.C.—This organisation for supplying wholesome food to the hungry was established in 1893, and during the first year of its existence distributed 50,000 dinners. Last year (to June 30th, 1896) 136,087 meals were provided. The meals are sold to the poor at 1d. each, except in destitute cases, when the use of tickets purchased by friends of the society is allowed. It is hoped in time to make each depôt self-supporting, but at present there is a loss of an average of ·831d. on each meal sold, so that subscribers are needed for the purpose of carrying on the work. Secretary and General Manager, Mr. A. C. Field.

**Orphan Working School,** Haverstock Hill, N.W., and Hornsey Rise, N. Offices, 73, Cheapside, E.C. Founded 1758.—This is a national and undenominational institution which maintains 500 children, varying in age from infancy to fourteen or (in special cases) fifteen years. It is greatly in want of funds at the present time, urgently needed to meet immediate and pressing liabilities. This orphanage is the oldest one of the kind in the metropolis, and a large percentage of scholars turn out satisfactorily. Secretary, Mr. Algernon C. P. Coote, M.A.

**Royal Albert Orphan Asylum,** Bagshot.—This institution affords a home and industrial training for about 200 fatherless children. No canvassing or sale of votes is permitted. Help is urgently needed, as the expenditure last year exceeded the income by some £650, and a curtailment of the work will be necessary unless funds are forthcoming. Secretary, Mr. H. W. Tatum. Office, 62, King William Street, E.C.

**Royal Sea Bathing Infirmary for Scrofula,** founded at Margate 1791.—This institution has been so badly supported that for some years past many of the wards have been closed. The efforts to remedy this state of affairs have recently been fairly successful, and by the end of this year it is hoped that only 80 of the 220 beds will be unavailable for patients. All information will be gladly given by the Secretary, Mr. A. Peirce, 30, Charing Cross Road, S.W. Treasurer, Mr. M. Biddulph, M.P.

**Surgical Aid Society,** Salisbury Square, E.C.—The annual subscriptions to this society have reached the sum of £6,985, and the total income last year was £11,807. The net expenditure amounted to £11,355, of which 83 per cent. was for actual relief. During the past year the large total of 21,513 appliances were given away. There is ample scope for very considerable extension of these benefits, and, therefore, the committee earnestly appeal for contributions. Secretary, Mr. Richard C. Tresidder.

**Thames Church Mission,** 31, New Bridge Street, E.C.—Most people have a warm corner in their hearts for sailors and seamen generally. Money to carry on its helpful work amongst the seafaring comers and goers on the Thames is much needed by this society. Secretary, Mr. F. Penfold.

**The Church of England Scripture Readers' Association.**—The society employs 130 readers who, visiting amongst the very poorest of the population in the metropolis under the direction of the parochial clergy, have much opportunity for doing good. A special effort is necessary to remove a deficiency on the accounts of the society, and thus enable it to continue the work. Secretary, Mr. Martin Tilby. Offices, 56, Haymarket, S.W.

## Figures and Tables

**Figure f1:**
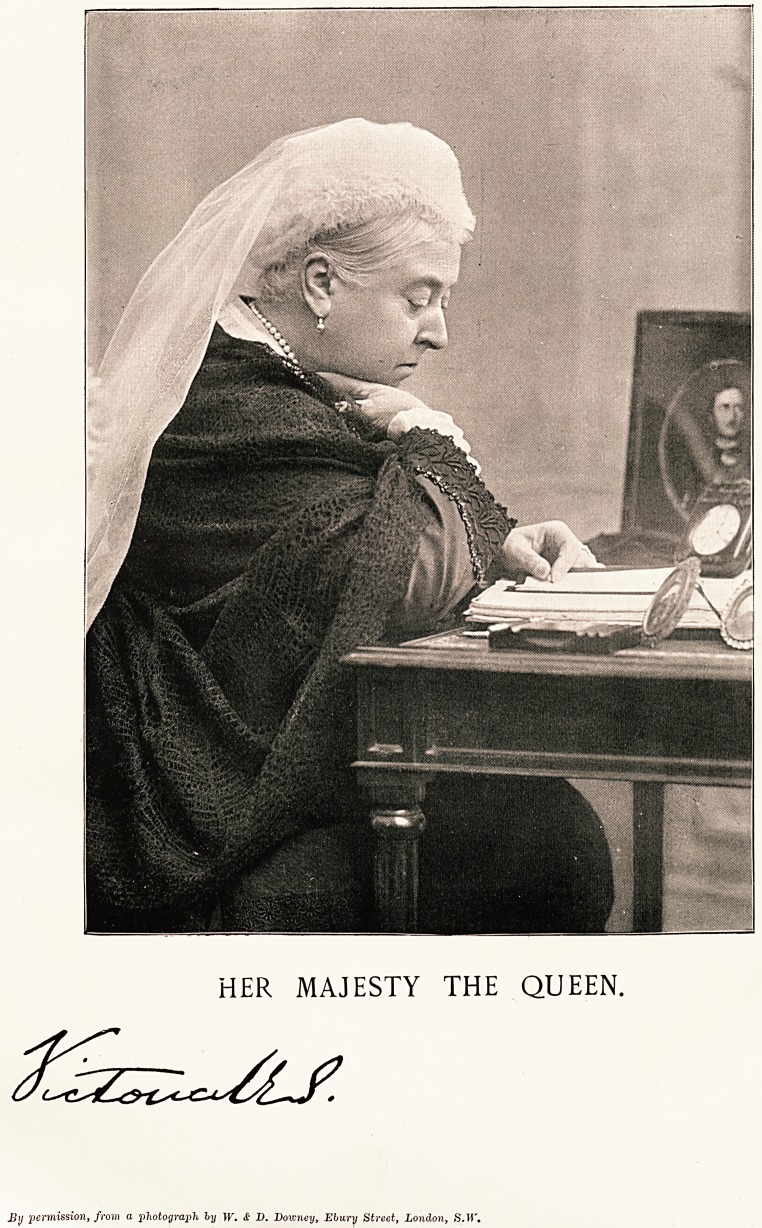


**Figure f2:**
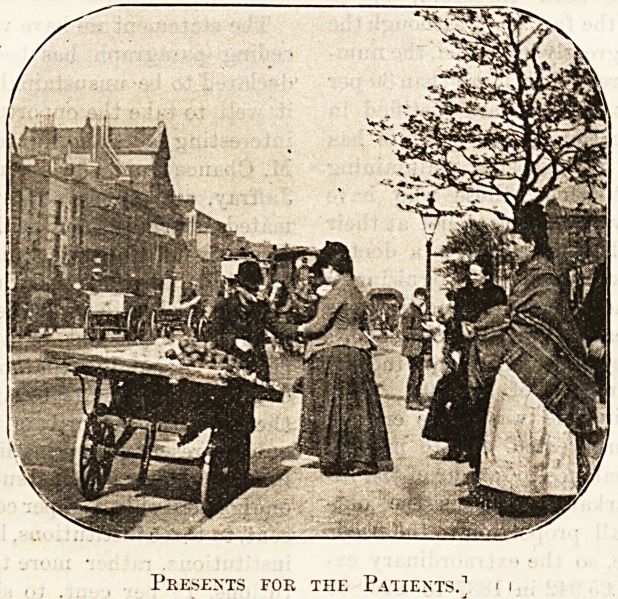


**Figure f3:**
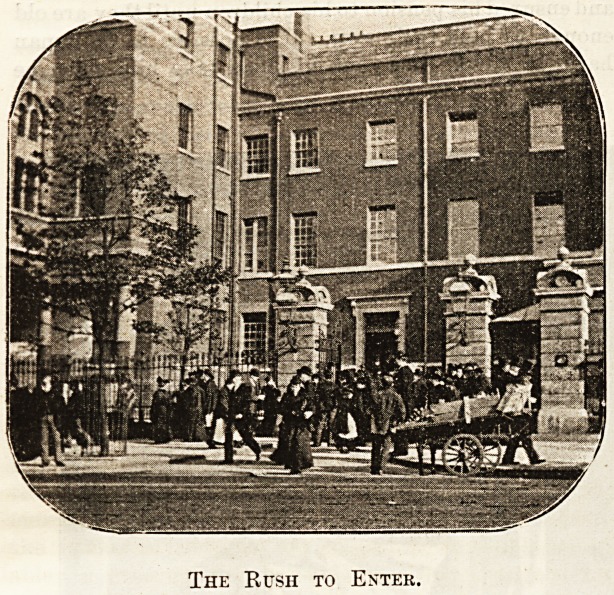


**Figure f4:**
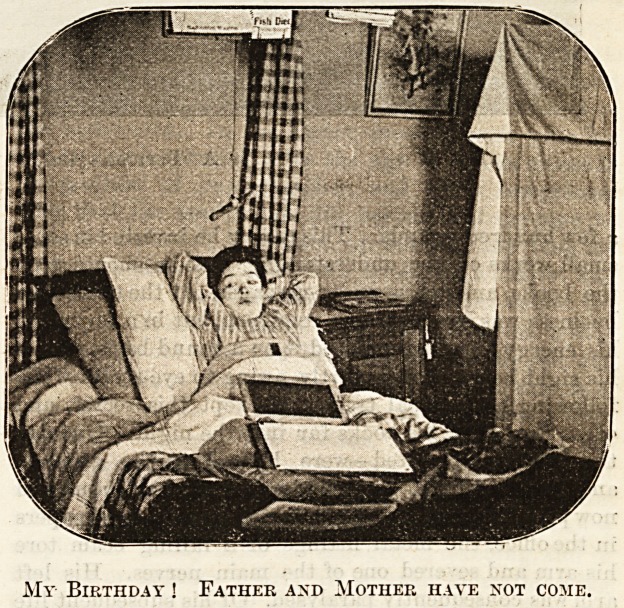


**Figure f5:**
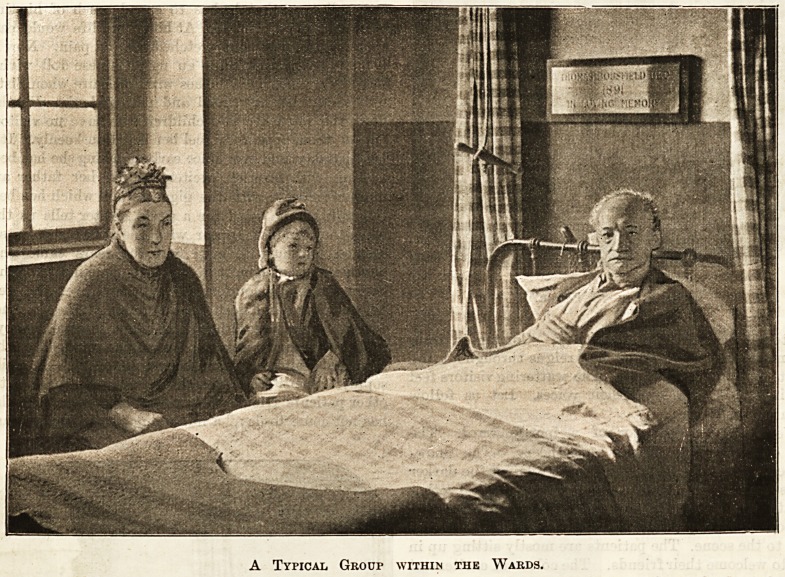


**Figure f6:**
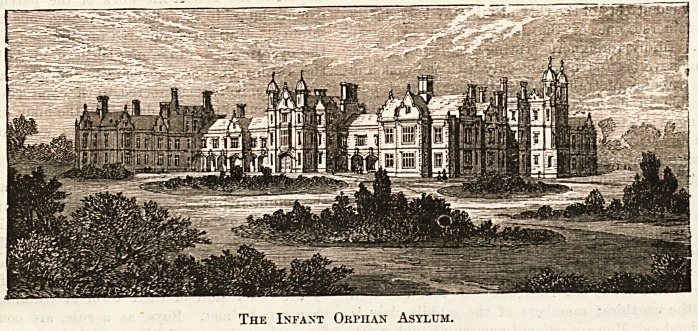


**Figure f7:**